# Impact of Conditional Cash Transfers on Maternal and Newborn Health

**Published:** 2013-12

**Authors:** Amanda Glassman, Denizhan Duran, Lisa Fleisher, Daniel Singer, Rachel Sturke, Gustavo Angeles, Jodi Charles, Bob Emrey, Joanne Gleason, Winnie Mwebsa, Kelly Saldana, Kristina Yarrow, Marge Koblinsky

**Affiliations:** ^1^Center for Global Development, 2055 L street NW (fifth floor), Washington, DC 20036, USA; ^2^United States Agency for International Development; ^3^Eunice Kennedy Shriver National Institute of Child Health and Human Development; ^4^National Institutes of Health; ^5^University of North Carolina–Chapel Hill; ^6^Population Council; ^7^Save the Children

**Keywords:** Conditional cash transfers, Global health, Incentives, Maternal health, Millennium Development Goals, Newborn health, Social protection

## Abstract

Maternal and newborn health (MNH) is a high priority for global health and is included among the Millennium Development Goals (MDGs). However, the slow decline in maternal and newborn mortality jeopardizes achievements of the targets of MDGs. According to UNICEF, 60 million women give birth outside of health facilities, and family planning needs are satisfied for only 50%. Further, skilled birth attendance and the use of antenatal care are most inequitably distributed in maternal and newborn health interventions in low- and middle-income countries. Conditional cash transfer (CCT) programmes have been shown to increase health service utilization among the poorest but little is written on the effects of such programmes on maternal and newborn health. We carried out a systematic review of studies on CCT that report maternal and newborn health outcomes, including studies from 8 countries. The CCT programmes have increased antenatal visits, skilled attendance at birth, delivery at a health facility, and tetanus toxoid vaccination for mothers and reduced the incidence of low birthweight. The programmes have not had a significant impact on fertility while the impact on maternal and newborn mortality has not been well-documented thus far. Given these positive effects, we make the case for further investment in CCT programmes for maternal and newborn health, noting gaps in knowledge and providing recommendations for better design and evaluation of such programmes. We recommend more rigorous impact evaluations that document impact pathways and take factors, such as cost-effectiveness, into account.

## INTRODUCTION

Given the slow decline in maternal and newborn mortality since 1990, the achievement of Millennium Development Goal 4 and 5—reducing infant mortality rate by two-thirds and maternal mortality rate by three-quarters from 1990 to 2015–is unlikely. Most of these deaths occur in the intrapartum and immediate postpartum period largely from preventable causes (1,2). Annually, about 60 million women give birth outside of health facilities, mainly at home and 52 million without a skilled birth attendant (3). Family planning needs are met for only about 50% of women (4), and total fertility rate (TFR) is still very high in low-income countries (5). Further, skilled birth attendance and the use of antenatal care are most inequitably distributed in 12 key maternal, newborn and child health interventions studied in low- and middle-income countries (LMICs), with poorer women facing higher barriers to access (4). The reasons behind the limited use of maternal health services by the poor are myriad and occur on both demand (households, women) and supply (provider) sides but a key demand-side obstacle relates to financial barriers (6).

Conditional cash transfer (CCT) is a type of demand-side programme that has been used in overcoming financial barriers to healthcare. CCT is a component of social programmes that condition regular cash payments to poor households on the use of certain health services and school attendance. These programmes have two main objectives: first, to provide a safety net to smooth the consumption of the extremely poor (alleviating short-term poverty) and, second, to increase the human capital investment of poor households (alleviating long-term poverty). Payments are usually provided to women, and compliance with conditions is verified by the programme. Transfers are generally sized to close the gap between average consumption in the bottom quintile of the income distribution and the extreme poverty line. Initially based in Latin America (Mexico, Brazil, Nicaragua, Honduras), the CCT programmes now operate around the world and are regarded as successful social protection strategies, given their impact in increasing investments to human capital (7). Most CCT programmes are broad, aiming at alleviating poverty and increasing human capital through transfers that are conditioned on a combination of school attendance, use of well-child visits, vaccination, and/or use of nutritional supplements. However, ‘narrow’ CCT programmes that transfer cash only for the utilization of specific services are becoming more common; for example, India's *Janani Suraksha Yojana* (JSY) and Nepal's Safe Delivery Incentive Program (SDIP) specifically target improvements of maternal and newborn health.

However, unlike the ‘broad’ CCT, the ‘narrow’ programmes, like JSY and SDIP, do not always or only target low-income groups. In JSY, a mix of geographical and income-targeting is used in inducing pregnant women to seek care while, in Nepal, cash incentives are offered to all pregnant women (8).

**Figure 1.**
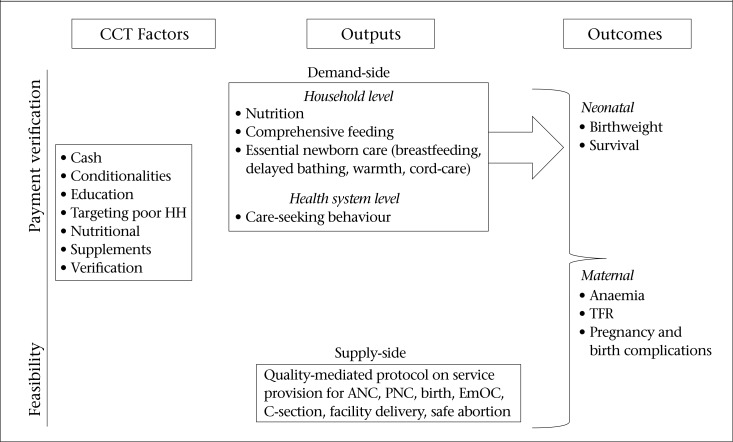
Conceptual framework of CCT programmes

Although programmes differ in their specific design features, CCT programmes usually share the following key features (Figure 1).

- Cash transfers that are conditioned on the utilization of a service as mandated under the programme, i.e. health, education, and nutrition
- Health information, education and communication (IEC) efforts
- Ex-ante identification (targeting) of recipient communities or households, using a variety of criteria
- Verification of compliance with conditions.

Figure 1 demonstrates a conceptual framework of CCT programmes, which can be associated with MNH outputs, outcomes, and impact from both demand and supply sides. On the demand side, as income increases via the cash transfer and as knowledge is enhanced via education/IEC interventions, household-level outputs, such as improved nutrition and feeding, may be affected by CCT as could better newborn care, such as exclusive breastfeeding, delayed bathing, warmth, and cord-care. At the health system level, demand-side outputs, such as utilization of specific services, including antenatal care (ANC), facilities for birth, and skilled birth attendance, may be affected via both reduction in costs associated with care-seeking and increased knowledge resulting from education/IEC programme components. Increased demand for services may also trigger improvements in the supply of services via greater provider responsiveness (e.g. less absenteeism).

To support such improvements, some conditional cash transfer programmes include components that support the supply side, such as strengthening health services in programme areas. In its initial phase, Nicaragua's RPS, for example, contracted non-governmental organizations to provide an essential package of services to CCT beneficiaries and non-beneficiaries in intervention communities (9), Similarly, India's JSY programme has a supply-side component, including incentive payments to community-level health workers for bringing pregnant women to a designated facility for delivery (10). Together, demand-side and supply-side outputs mediated by contextual factors are expected to jointly generate improved newborn outcomes and impact, such as higher birthweights and survival (both in perinatal and neonatal periods). Improved maternal health outcomes could include reduced anaemia and complications during pregnancy and birth, and potentially maternal mortality.

Although few CCT programmes have explicitly targeted the improvement of maternal and newborn health, many of the ‘broad’ programmes included conditionality, associated supply-side strengthening, and/or educational talks relating to MNH, and many impact evaluations have measured the effects of CCT on MNH interventions and outcomes. Further, as in programmes that relax a household's budget constraint, CCT can be expected to affect household spending choices in general, with the potential to improve MNH. Other systematic reviews have documented the effects of CCT on childcare utilization and nutritional status (11); yet, no paper has directly reviewed the evidence on impact of CCT on MNH or the use of appropriate MNH services.

This paper seeks to fill this gap, setting out the hypothesized channels through which impact of CCT on MNH may occur, synthesizing the empirical evidence on the impact of CCT on MNH interventions and outcomes, discussing issues emerging from synthesis of the evidence, and providing recommendations for the future. Specifically, we look into the effect of CCT on maternal and neonatal health outcomes, the use or provision of maternal health services, or into care-seeking behaviour by women.

The literature synthesized in the paper was gathered for an Evidence Summit convened by USAID in April 2012. The rationale for the Evidence Summit was to evaluate the existing evidence on answers to critical questions from academia, other US agencies, multilaterals, and other countries. The characteristics of the CCT programmes that have MNH outcomes are reported in Table 1 and 2.

## MATERIALS AND METHODS

Our initial search “conditional cash transfers and maternal health” resulted in 5,800 results on Google Scholar, of which 470 documents remained after duplicates were removed. A call for relevant papers led to 26 additional documents. A total of 65 documents remained after the screen was applied, categorized according to financial incentive and outcome(s) reported. Of these, 9 articles applied to CCT.

In addition, a search for non-financial strategies to increasing care-seeking for MNH services was conducted using the search terms, ‘care-seeking and maternal’, ‘care seeking and newborn’, ‘care-seeking and postpartum family planning’ through search engines Medline and Cochrane Collection; 72 hits were received and abstracts reviewed. Final articles selected numbered 24 and included primarily in Cochrane reviews, systematic reviews, with single published papers that explored specific issues of interest (e.g. birth preparedness complication readiness).

Although we do not have more than one study for each programme, the studies included well-structured impact evaluations with experimental or quasi-experimental designs, with output measures that are relatively comparable and consistent across different studies.

Of the CCT studies that report rigorously-calculated impacts, outcome variables that were common across at least two studies were identified; baseline values, effect-sizes (reported as the average treatment effect, or the difference between treatment and control groups), standard errors, significance, sample-sizes, and scope of the programme defined as the ratio of beneficiaries over the total population are reported.

**Figure 2(A-H).**
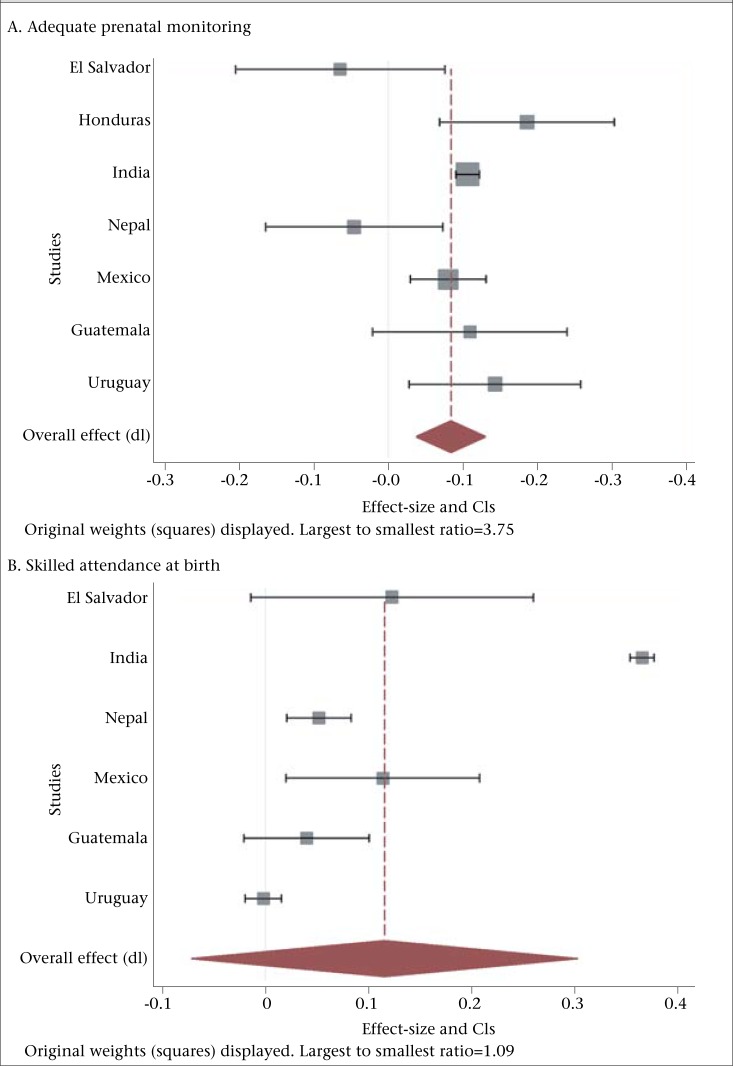

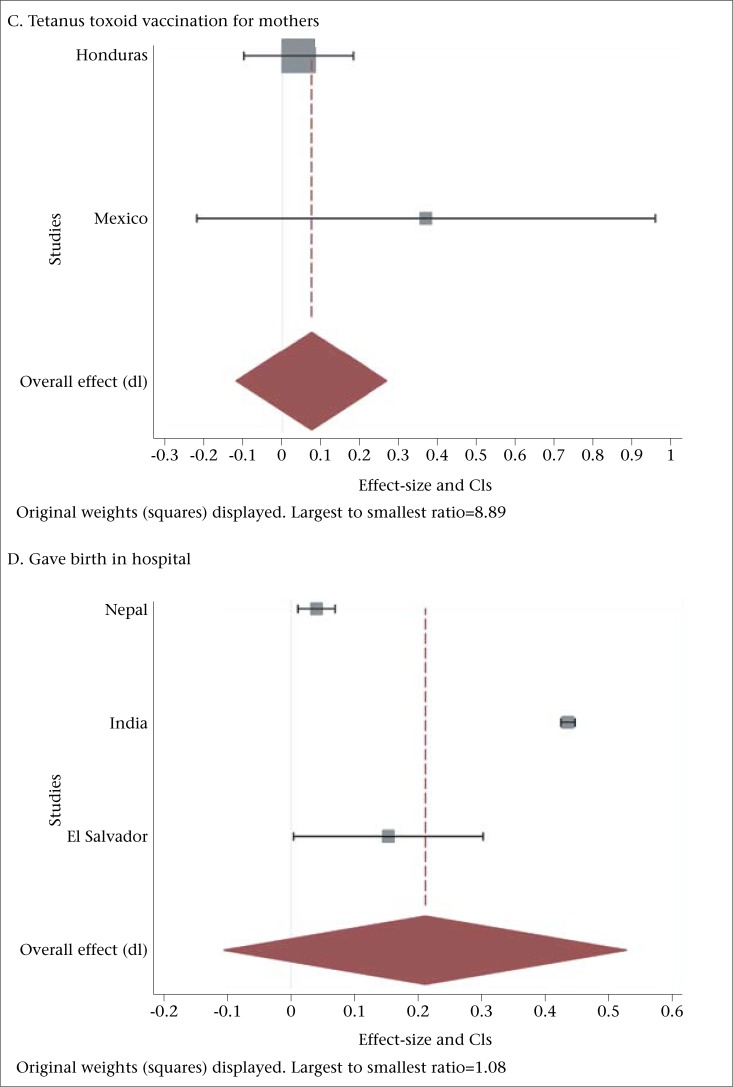

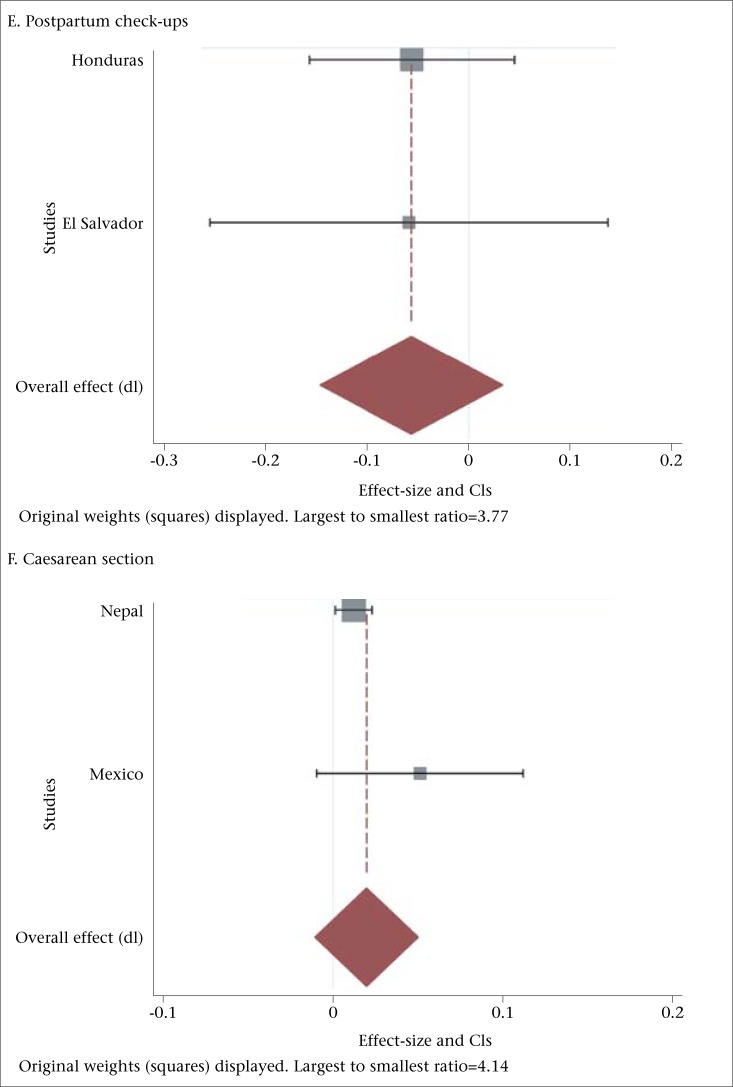

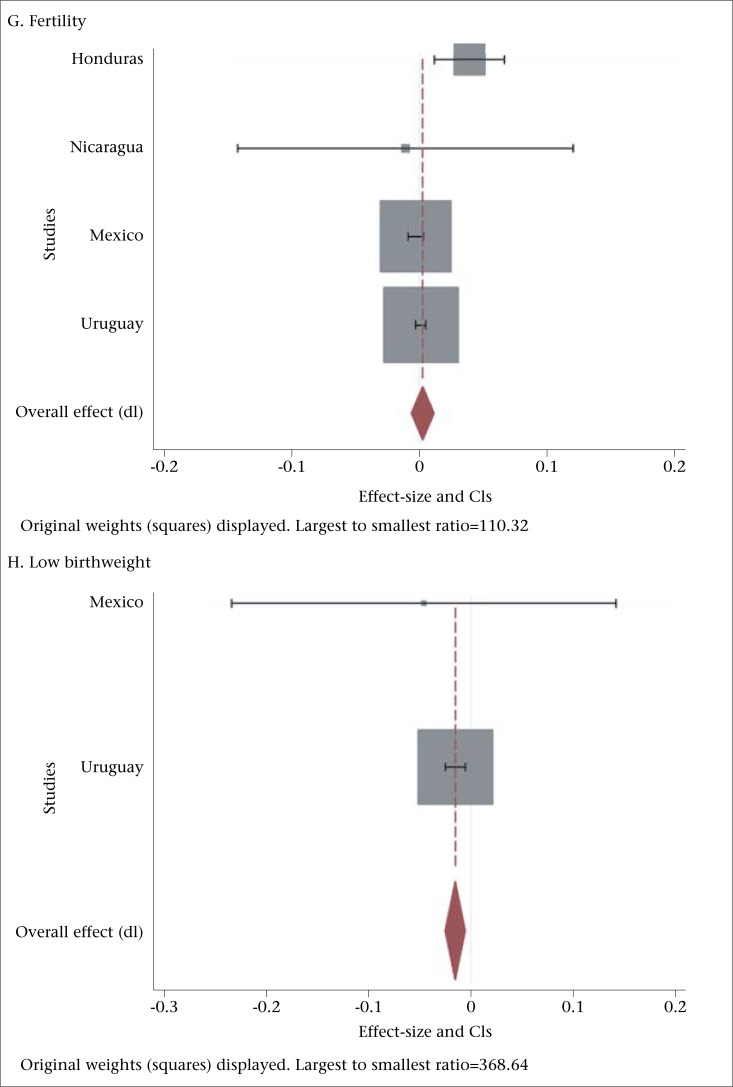
Forest plots on selected parameters of maternal and neonatal health

Forest plots are used in depicting effect-sizes and pooled estimates. To mitigate non-comparability, we use a DerSimonian–Laird random-effects model, a widely-used method to construct forest plots. Assuming heterogeneity between studies, this method uses a non-iterative method to estimate the inter-study treatment effect variance, without making any assumptions regarding the distribution of within-study or between-study effects. These estimations are generated with the metaan function on STATA 12. The final forest plots show individual effect-sizes with confidence intervals as well as a final average effect-size; the size of the boxes shows the significance of the effect where larger boxes indicate a wider range and larger confidence intervals. We report pooled average effects only when two or more studies are available. Table 3 summarizes MNH indicators, and in Figure 2 (A to H), we show forest plots for each outcome variable across available studies. In Table 4, we also provide a table of effect-size and confidence intervals for all MNH-relevant variables reported in the studies.

**Table 1.** Effect-sizes of CCT programmes for maternal and neonatal health

Study and country	Baseline	Effect-size	Standard error	Significance	Sample-size	% of population as beneficiary
Adequate prenatal monitoring^1^						
de Brauw and Peterman (2011) (El Salvador) (13)	0.768	-0.065	0.072	NS	494	0.131
Morris, Flores, Olinto, and Medina (2004) (Honduras) (12)	0.379	0.187	0.060	***	313	0.150
Lim *et al.* (2010) (India) (15)	0.536	0.107	0.008	***	182,869	0.100
Powell-Jackson *et al.* (2010) (Nepal) (14)	***1.235***	-0.046	0.061	NS	5,901	No targeting
Barber and Gertler (2009) (Mexico) (16)	0.612	0.081	0.026	***	892	0.180
IDB; Gutierrez *et al.* (2011) (Guatemala) (35)	2.69	0.11	0.067	**	1,163	0.057
Amarante *et al.* (2011) (Uruguay) (17)	***6.53***	0.144	0.059	**	67,863	0.100
Birth attended by skilled personnel						
de Brauw and Peterman (2011) (El Salvador) (13)	0.738	0.123	0.070	*	536	0.131
Lim *et al.* (2010) (India) (15)	0.593	0.366	0.006	***	182,869	0.100
Powell-Jackson *et al.* (2010) (Nepal) (14)	0.225	0.052	0.016	***	5,901	No targeting
Urquieta *et al.* (2009) (Mexico) (36)	0.305	0.114	0.048	**	860	0.180
IDB; Gutierrez *et al.* (2011) (Guatemala) (35)	0.105	0.04	0.031	*	1,006	0.057
Amarante *et al.* (2011) (Uruguay) (17)	0.49	-0.002	0.009	NS	68,855	
Tetanus toxoid for mothers						
Morris, Flores, Olinto, and Medina (2004) (Honduras) (12)	0.563	0.042	0.071	NS	313	0.150
Barber and Gertler (2009) (Mexico) (16)	0.924	0.368	0.300	NS	892	0.180
Gave birth in hospital						
Powell-Jackson *et al.* (2010) (Nepal) (14)	0.106	0.04	0.015	***	5,901	No targeting
Lim *et al.* (2010) (India) (15)	0.541	0.435	0.006	***	182,869	0.100
de Brauw and Peterman (2011) (El Salvador) (13)	0.733	0.153	0.076	*	530	0.131
Postpartum check-ups/visits after birth						
Morris, Flores, Olinto, and Medina (2004) (Honduras)^2^ (12)	0.178	-0.056	0.052	NS	311	0.150
de Brauw and Peterman (2011) (El Salvador) (13)	0.259	-0.059	0.100	NS	478	0.131
Contraceptives						
Feldman *et al.* (2009) (Mexico) (37)	0.37	0.16	0.097	**	16,462	0.180
Lamadrid-Figueroa *et al.* (2010) (Mexico) (20)	0.39	0.049	0.036	NS	2,239	0.180
Caesarean section						
Powell-Jackson *et al.* (2010) (Nepal) (14)	0.025	0.012	0.006	*	5,901	No targeting
Barber and Gertler (2009) (Mexico) (16)	0.145	0.051	0.031	**	979	0.180
Fertility						
Stecklov *et al.* (2007) (Nicaragua) (22)	0.108	-0.011	0.067	NS	4,885	0.027
Stecklov *et al.* (2007) (Mexico) (22)	0.179	-0.003	0.003	NS	17,634	0.180
Amarante *et al.* (2011) (Uruguay) (17)	NA	0.001	0.000	***	1,037,793	0.100
Low birthweight						
Barber and Gertler (2009) (Mexico) (16)	NA	-0.046	0.096	**	804	0.180
Amarante *et al.* (2011) (Uruguay) (17)	0.102	-0.015	0.005	***	68,858	0.100

*Significant at 10% level;

**Significant at 5% level;

***Significant at 1% level;

^1^Defined as 5 or more visits in every paper, except for India where it is defined as 3 or more visits;

^2^Defined as a 10-day postpartum check-up;

NA=Not available;

NS=Not significant;

Numbers in bold italics are number of visits;

other numbers are percentages of the population who have received adequate prenatal monitoring

## RESULTS

We describe the results of qualifying CCT studies on various MNH indicators summarized in Table 3 for how the studies define these terms. These same results are depicted graphically in forest plots in Figure 2 (A to H). Results on MNH mortality and service utilization and outcomes from CCT programmes are also discussed.

Almost every study points to a significant increase in utilization of different MNH services, and the results are consistent across different countries. However, studies differ in terms of what services they target. The majority of the programmes reviewed report an increase in adequate prenatal monitoring, ranging from an 8 percentage point difference in Mexico to a 19 percentage point increase in Honduras. The programmes in Honduras, India, and Uruguay are the only ones that included a specific conditionality relating to the use of antenatal care; other programmes (in El Salvador, Mexico, and Guatemala) only required preventive healthcare utilization by children while the remaining programmes only conditioned facility-based births (12). Two programmes—in El Salvador and Nepal—reported a small decline in the average number of antenatal visits but these results were insignificant (13,14). The Indian JSY programme conditioned three or more visits and reports an 11 percentage points increase.

Another common service output reported is related to births attended by skilled personnel, where every study reported positive and significant effects, from a low of 4 percentage points difference between beneficiaries and non-beneficiaries in Guatemala to a high of 37 percentage points difference in India (15). Similarly, the three studies that report births in health facilities show positive and significant effects and effect-sizes much larger than those reported for other outputs. In Nepal, there was a 4 percentage point difference between beneficiaries and non-beneficiaries (14) while Lim *et al*. reported a 43.5 percentage points difference in India (15).

**Table 2.** Details of cash transfer programmes by country

Country	Name of programme	Year started	Targeting and eligibility	Number of beneficiaries	Health conditions	Education conditions	Verification	Supply-side conditions and additional benefits	Type of evaluation	Reference(s)
El Salvador	Red Solidaria	2005	Geographic /Proxy means-testing	100,000 households	Compliance with immunization and regular health and nutrition monitoring	Primary school enrollment/ 80% school attendance (5-15 years)	Health and education personnel provide information to NGO	Yes; supply-side component to strengthen basic health and nutrition services in the targeted areas	Regression discontinuity design, differences-in-differences	de Brauw and Peterman (2011)
Guatemala	Mi Familia Progresa	2008	Geographic /Proxy means-testing	250,000 households	Regular health visits for children [0-16 year(s)] and pregnant women	90% of school attendance	Not fully implemented	No	Differences-in-differences	Gutierrez *et al.* (2011)
Honduras	Programa de Asignación Familiar	1998	Geographic /Proxy means-testing	240,000 households	Compliance with required frequency of health centre visits; children attend growth monitoring; pregnant women receive at least 4 ANC visits	School enrollment/ 85% school attendance	None	Yes; promote access to an integrated package of services, including nutrition, healthcare, and basic services. Improve quality of facilities due to service-level package	Cluster-randomized trial, with a pre-test and post-test cross-sectional design	Morris, Flores, Olinto, and Medina (2004)
India	*Janani Suraksha Yojana*	2005	Poverty-line estimates	9,500,000 women	Delivery in health facility, antenatal check-ups	None	Community-level health workers	Yes; payments to ASHAs who identify pregnant women and help them get to a facility	Matching, with versus without comparison, differences-in-differences	Lim *et al.* (2010)
Mexico	*Oportunidades* (formerly PROGRESA)	1997	Geographic/Proxy means-testing	5,000,000 households	Children <2 years fully immunized and undergo growth monitoring. Prenatal visits, breastfeeding, physical check-ups	80% school attendance (monthly), and 93% (annually)/ Completion of middle school/ Completion of grade 12 before age 22 years	Programme state coordination agency	No	Regression discontinuity design, differences- in-differences	Urquieta *et al.* (2009); Stecklov *et al.* (2007); Sosa-Rubi *et al.* (2011); Barber and Gertler (2009); Feldman *et al.* (2009); Lamadrid-Figueroa *et al.* (2010)
Nepal	Safe Delivery Incentive Program (SDIP)	2005	All women	100,000 women	Deliver in a public health facility and had no more than two living children or an obstetric complication. Skilled attendance at birth	None	Deliver in health facility	Yes; provider incentives (US$ 5 for each delivery attended)	Propensity score matching	Powell-Jackson *et al.* (2009); Powell-Jackson *et al.* (2011)
Nicaragua	Red de Protección Social	2000	Geographic	3,000 households	Bimonthly health education workshops/Monthly healthcare visits (aged 0-2) or bimonthly (aged 3-5)/Adequate weight gain and up-to-date vaccinations (aged 0-5 years)	School enrollment in grades 1-4 (7-13 years)/ 85% school attendance (every 2 months)/Grade promotion at end of every year	Forms (confirmed by service providers and put into information system)	Yes; health education workshops every 2 months, child growth and monitoring, provision of antiparasite medicine, vaccinations, teacher transfer	Differences-in-differences	Stecklov *et al.* (2007)
Uruguay	Plan de Atención Nacional a la Emergencia Social	2005	Poverty-line estimates	102,000 individuals	Regular ANC health visits for pregnant women and children	NA	Visits; although not rigorously enforced	No	Regression discontinuity design, differences- in-differences	Amarante *et al.* (2011)

ANC=Antenatal care;

NA=Not available

Two studies reported the effect of CCT on caesarean section at the last birth among beneficiaries and non-beneficiaries. Both studies reported positive and significant effects; in Nepal, there was one percentage point difference between the intervention and control groups (14) while this difference was 5 percentage points in Mexico (16)–the overall effect is a 2% increase. However, the baseline rate in Mexico among beneficiaries and their controls was already 15% of all births–the top of the WHO-recommended level; so, it is not possible to interpret whether this increase is consistent with MNH recommendations for better outcomes.

Two studies analyze the effect of CCT on the incidence of low birthweight in Mexico and Uruguay (16,17). Both studies report a small but significant decline in the incidence of low birthweight: in Mexico, the proportion of infants born with low birthweight declined by 4.6% and, in Uruguay, by 1.5% [Overall effects refer to the effect-sizes that are calculated in the DerSimonian-Laird random effect models (see Table 3 for weights assigned by this method)]. However, an unpublished job market paper on Indonesia found that the CCT programme did not have an impact on low birthweight or other birth outcomes (18).

The CCT programmes have failed to generate impact for some variables. Two studies reported the impact of CCT on the probability that a mother would receive a tetanus toxoid vaccination, an intervention that is essential to ensure survival of both mother and the baby in LMICs, especially where there is a large share of home births (12,19) but these effects were not statistically significant. Similarly, the CCT programmes have had no effect on postpartum visits, which are considered critical for both mother and the newborn, especially in the immediate 48 hours through the first week following birth (12,13).

Only one programme–Mexico's *Oportunidades*–reported on the use of contraceptive, finding that beneficiaries were 16 percentage points more likely to use a modern contraceptive method than non-beneficiaries. Another analysis of Mexico's *Oportunidades* looks at heterogeneous effects, finding a small and insignificant effect on the use of contraceptive (20). There are also results on risky sexual behaviour from both *Oportunidades* and the CCT in Malawi: the Malawi study found that those in the CCT programme were 0.29 times less likely to test HIV-positive (21).

**Table 3.** Effect-sizes by outcome and country

Outcome	Country	Effect-size	Lower CI, 95%	Upper CI, 95%	% weight
Adequate prenatal monitoring	El Salvador	-0.065	-0.206	0.076	7.96
	Honduras	0.187	0.069	0.305	10.29
	India	0.107	0.091	0.123	29.86
	Nepal	-0.046	-0.166	0.074	10.07
	Mexico	0.081	0.03	0.132	22.47
	Guatemala	0.11	-0.021	0.241	8.84
	Uruguay	0.144	0.028	0.26	10.52
	Overall effect (dl)	0.084	0.038	0.131	100
Birth attended by skilled personnel	El Salvador	0.123	-0.014	0.26	15.66
	India	0.366	0.354	0.378	17.08
	Nepal	0.052	0.021	0.083	17.01
	Mexico	0.114	0.02	0.208	16.39
	Guatemala	0.04	-0.021	0.101	16.79
	Uruguay	-0.002	-0.02	0.016	17.07
	Overall effect (dl)	0.116	-0.072	0.303	100
Tetanus toxoid for mother	Honduras	0.042	-0.098	0.182	89.89
	Mexico	0.368	-0.22	0.956	10.11
	Overall effect (dl)	0.075	-0.118	0.268	100
Mother gave birth in health facility	Nepal	0.04	0.011	0.069	34.08
	India	0.435	0.424	0.446	34.16
	El Salvador	0.153	0.004	0.302	31.76
	Overall effect (dl)	0.211	-0.105	0.527	100
Postpartum check-ups/visits after birth	Honduras	-0.056	-0.157	0.045	79.02
	El Salvador	-0.059	-0.255	0.137	20.98
	Overall effect (dl)	-0.057	-0.146	0.033	100
Caesarean section	Nepal	0.012	0.001	0.023	80.53
	Mexico	0.051	-0.01	0.112	19.47
	Overall effect (dl)	0.02	-0.011	0.05	100
Fertility	Honduras	0.039	0.012	0.066	8.48
	Nicaragua	-0.011	-0.142	0.12	0.44
	Mexico	-0.003	-0.009	0.003	42.93
	Uruguay	0.001	-0.003	0.005	48.16
	Overall effect (dl)	0.002	-0.006	0.011	100
Low birthweight	Mexico	-0.046	-0.234	0.142	0.27
	Uruguay	-0.015	-0.025	-0.005	99.73
	Overall effect (dl)	-0.015	-0.025	-0.005	100

Definitions of terms: *Low birthweight*=The papers cited here use the World Health Organization's definition of a newborn weighing less than 2,500 g (5.5 pounds); *Adequate antenatal monitoring*=Adequate antenatal monitoring is defined as 5 or more visits to a health facility for antenatal monitoring, except for the Indian study, which defines it as 3 or more visits to a health facility for antenatal monitoring; *Births attended by skilled personnel*=Skilled attendance at birth is defined as attendance by a doctor, an obstetrician/gynaecologist, a nurse or a midwife; *Births in health facility*=Studies define ‘facility’ differently; the El Salvador study includes births in a public or private facility and excludes births at health centres or mobile health clinics; the Indian study includes any kind of health facility; *Postpartum check-ups/visits*=A visit to a health facility within 10-14 days of giving birth; dl=Generated using a DerSimonian-Laird (dl) random-effects model

**Table 4.** Reviewed variables and effects by study

Study	Dependent variable	ES	95% CI
de Brauw and Peterman (2011) (El Salvador)	Adequate prenatal monitoring (5 or more visits)	-0.065	-0.059, 0.065
	Birth attended by skilled personnel	0.123	0.129, −0.006
	Gave birth in hospital	0.153	0.147, 0.159
	Mother went for postnatal check-up	-0.059	-0.050, −0.068
Morris, Flores, Olinto, and Medina (2004) (Honduras)	Adequate prenatal monitoring (5 or more visits)	0.187	0.074, 0.30
	Adequate prenatal monitoring (5 or more visits)	0.184	0.069, 0.299
	Adequate prenatal monitoring (5 or more visits)	0.132	-0.016, 0.28
	10-day postpartum check-up	-0.056	-0.157, 0.045
	10-day postpartum check-up	-0.057	-0.16, 0.045
	10-day postpartum check-up	0.012	-0.118, 0.143
	Child taken to health centre at least once in last 30 days	0.202	0.109, 0.296
	Child taken to health centre at least once in the last 30 days	0.149	0.056, 0.243
	Child taken to health centre at least once in the last 30 days	-0.018	-0.134, 0.098
	Tetanus toxoid for mothers	0.042	-0.097, 0.182
	Tetanus toxoid for mothers	0.081	-0.061, 0.222
	Tetanus toxoid for mothers	0.064	-0.116, 0.244
	Weighed in the last 30 days (mothers)	0.211	0.111, 0.311
	Weighed in the last 30 days (mothers)	0.176	0.075, 0.276
	Weighed in the last 30 days (mothers)	0.08	-0.044, 0.204
Barber and Gertler (2009) (Mexico)	Tetanus toxoid for mother	0.368	NA
	Average physical examination visits	0.059	NA
	Iron supplements	0.053	NA
	Average increase in prevention and case management	0.043	NA
	Prenatal procedures received	0.122	NA
Lim *et al.* (2010) (India)	Adequate prenatal monitoring (3 or more visits)	0.109	0.046, 0.172
	Birth attended by skilled personnel	0.393	0.337, 0.45
	In-facility births	0.492	0.432, 0.551
	Perinatal deaths (per 1,000 pregnancies)	-14.2	-31.0, 2.7
	Neonatal deaths (per 1,000 livebirths)	-6.2	-20.4, 8.1
	Maternal deaths (per 100,000 livebirths)	-100.5	-582.2, 381.2
Ozer *et al.* (2011) (Mexico)	Full depression scale (0-60)	-1.71	-2.46, −0.96
Powell-Jackson *et al.* (2011) (Nepal)	Delivery at facility	0.04	0.05,0.31
	Birth attended by skilled personnel	0.052	0.06, 0.28
Powell-Jackson *et al.* (2009) (Nepal)	Birth attended by skilled personnel	0.023	-0.082, 0.129
	Number of antenatal care visits	0.031	0.008, 0.054
	Neonatal mortality	-0.0004	0,0
	Delivery at home	-0.042	-0.329, 0.245
	Delivery at government facility	0.026	0.168, −0.116
	Delivery at private facility	0.002	0.007, −0.003
	Delivery with a health worker	0.044	0.342, −0.254
	Delivery by caesarean section	-0.001	0.0, −0.002
Sosa-Rubi *et al*. (2011) (Mexico)	Antenatal visits	0.021	
Baird *et al.* (2011) (Malawi)	Teenage pregnancy (conditional transfer)	0.029	0.027
	Teenage pregnancy (unconditional transfer)	-0.067	0.024
Stecklov *et al.* (2007) (Latin America)	Fertility; controlled for education, age, household, wealth (Honduras)	0.039	0.002
	Fertility; controlled for education, age, household, wealth (Nicaragua)	0.009	0.565
	Fertility; controlled for education, age, household, wealth (Mexico)	-0.003	0.852
Urquieta *et al.* (2009) (Mexico)	Skilled attendance at delivery	0.028	0.027
Alam *et al.* (2010) (Pakistan)	Probability of marriage	0.0082	0.008
	Age at marriage	1.46	0.621
	Probability of giving birth	-0.0808	0.172
	Number of children	-0.329	0.181
*Oportunidades*, Official evaluation (Mexico)	Maternal anaemia for women of childbearing age, urban	0.003	
	Maternal anaemia for women of childbearing age, rural	-0.014	
IDB; Gutierrez *et al.* (2011) (Guatemala)	Folic acid supplement	0.07	*
	Iron supplement	0.1	**
	Number of prenatal visits at health centres	0.11	**
	Skilled attendance at delivery	0.01	
Baird *et al.* (2009) (Malawi)	Risky sexual activity	-0.159	
	Sexual activity (number of partners)	-0.036	
	Teenage pregnancy	-0.051	**
Barber (2009) (Mexico)	Caesarean section rate	0.0508	**

ES=Effect-size

In terms of impact, studies report on fertility and maternal mortality, with mixed results. A study looking at the changes in fertility rates from Honduras, Nicaragua, Mexico, and Uruguay reports impact on the age-specific and total fertility rates, ranging from a 4% increase in Honduras to a 1% decrease in Nicaragua (22). A study on JSY finds that fertility increased by 1.1 percentage points but reports non-comparable outcomes and is, therefore, not included in Table 2 (23) [The study by Mazumdar *et al*. (23) measures fertility by assuming that respondents are pregnant for 6 months when they respond to the questionnaire whereas the study by Stecklov (22) reports fertility based on baseline surveys that were conducted before the programme, and it estimates fertility, using differences-in-differences].

**Box.** India's *Janani Suraksha Yojana* (JSY) programme: Issues with impact evaluations

India's JSY is the largest CCT programme in the world and specifically targets MNH. This, coupled with the fact that India has the highest number of maternal deaths in the world, makes JSY's evaluation extremely important. The study by Lim *et al*. (15), the only published impact evaluation of the JSY programme to date (between 2002-2004 and 2007-2008), reports positive results for service uptake as well as neonatal mortality.

However, new studies challenge some of these findings. An unpublished evaluation by Mazumdar, Mills, and Powell-Jackson (23) finds similar results for increases in facility-based deliveries in JSY, also reporting that the programme was more effective for less educated, poor and ethnically-marginalized women. The study also finds increases in breastfeeding and less use of private healthcare providers. Going beyond the positive impact on service uptake, however, the study by Mazumdar *et al*. finds that JSY increases fertility and does not have an effect on antenatal care or neonatal mortality.

Other critics of the programme point to gaps in the evaluation. A letter published in *The Lancet* by Das *et al*. points to problems in the enforcement of conditions, inconsistencies in the implementation of the programme between states and problems in recording programme enrollment status due to an ambiguous question in the household survey (25). Beyond these concerns, a process evaluation of the programme by Devadasan *et al*. (26) raised issues of women receiving only a portion of the promised cash transfers as well as the transfer going to women who delivered at home.

These concerns about the world's largest CCT programme show the need for a systematized, more rigorous design for impact evaluation as well as more attention to process evaluation.

Three studies look at maternal mortality measures. The official evaluation of *Oportunidades* reports an 11% decline in maternal mortality in regions that had at least one locality incorporated in the *Oportunidades* programme (RR 0.89; CI 0.82-0.95) (24). Lim *et al*. report large declines in perinatal and neonatal deaths associated with India's JSY, although findings for maternal death were insignificant (15). Powell-Jackson *et al*. (2010) report a very small and insignificant decline in neonatal mortality in Nepal (14). However, given the issues in data collection, estimating impact on maternal mortality is prone to measurement errors and underestimation; so, we do not aggregate these results (see **Box** on criticisms regarding the JSY impact evaluation).

This review of effect-sizes suggests that CCT can reduce barriers to MNH service utilization, such as prenatal monitoring, skilled attendance at birth, and the use of facility for birth. Further, CCT may have an impact on the incidence of low birthweight as well as the more distal outcomes of fertility and mortality. However, it is difficult to empirically document the specific causal pathways that link CCT design features to impact as outlined in Figure 1 and in the text, given that evaluations were not designed to measure the effects of these pathways. As a result, these channels of impact remain hypotheses rather than well-substantiated relationships.

## DISCUSSION

From a programmatic standpoint, the potential impact of a CCT depends not only on design features of a programme but also on a range of contextual factors. In addition to income, cost and knowledge, care-seeking and health outcomes are also determined by the interplay of social, cultural and health system factors. As these factors vary by context, a CCT that is successful in one context may be unsuccessful in another, with the difference attributable to factors that are not typically assessed during a programme evaluation, such as cultural factors or supply-side constraints.

Some contextual enabling factors underpinning the effectiveness of CCTs are macro-economic stability, good infrastructure, strong information systems, and targeting mechanisms. For example, poor infrastructure would inhibit transportation to facilities, and low quality of care would constrain demand for MNH services. Most of these factors, however, have not been addressed: for instance, most evaluations do not assess or report supply-side constraints, such as quality of care. Yet, CCT do have potential to improve quality of care: for example, Barber and Gertler report positive effects of Mexico's *Oportunidades* programme on the number of prenatal procedures recommended by the Ministry of Health, provided during antenatal visits as well as the number of iron supplements provided. It is likely that the quality and availability of supply-side efforts (e.g. skilled care, emergency care facilities) have major impact, and there is some relationship between strengthening supply as part of a CCT and programme impact. Programmes in some states of India, Nicaragua, and Honduras included a supply-side component and, with the exception of Honduras for reasons relating to study design, report large positive and significant results. However, this issue has not been examined directly, and there are no other examples.

Another important dimension of CCT is the sustainability of the behaviour change desired and whether and how long improved behaviour relies on the existence of the financial incentive. As CCT programmmes are relatively new instruments for change, such behaviour change regarding the use of maternal health services has not been examined. Some evidence suggests that there can be a ‘learning effect’ whereby women with greater exposure time to a CCT programme engage in greater utilization of maternal health services. For example, an increase in the last delivery attended by a physician/nurse versus a traditional midwife in Mexico was reported, although the CCT only conditions the use of adequate antenatal care, not facility-based delivery, or the use of a skilled birth attendant (27). These studies do not, however, examine effect of the absence of the financial incentive on behaviour. More robust research in this area is needed to provide the evidence on sustainability of behaviour changes.

Going forward, it is important to consider the definition of outcome indicators, which currently vary across studies and should be standardized. For instance, “adequate prenatal care” means different things in different settings, and it was always defined, with the exception of Mexico and Guatemala, as number of prenatal visits. There is no evidence of the positive impact of the number of ANC visits on better health outcomes. Similarly, postpartum visits were defined as a 10-day postpartum check-up in Honduras; it is not clear whether a 10-day postpartum check-up would be a beneficial intervention.

The final issue to consider is the cost-effectiveness of these programmes, particularly compared to non-financial incentives. The CCT programmes are not directly comparable with non-financial demand-side programmes as the latter are typically small-scale and carried out by non-governmental organizations. Certain non-financial interventions, such as maternal waiting homes, integration of traditional birth attendants, birth preparedness, and complication readiness, community referrals, transport systems, and cellphone technologies to increase the use of skilled obstetric care, are promising but require more rigorous evaluation (28-31). Cost data, in particular, are weak and need to be reported not only for the interventions themselves but also for a standard outcome (such as DALYs) so that different interventions can be compared in terms of their cost-effectiveness.

### Conclusions and recommendations

Conditional cash transfer programmes are increasingly being adopted and scaled in developing countries, particularly programmes that target specific outcomes relating to maternal health, sexual behaviours, and/or vaccination practices. The CCT programmes are particularly gaining popularity in sub-Saharan Africa where 18 countries are implementing conditional cash transfer programmes (32), of which 3 have MNH-related requirements (Eritrea, Mozambique, and Senegal). Similarly, there are ongoing evaluations of CCT programmes for maternal health in Afghanistan, Bolivia, and the Philippines, and these evaluations can help us understand linkages between transfers, conditionality, utilization, and outcomes (33,34).

Our review of programmes finds that CCTs have increased the uptake of MNH services, especially skilled attendance at delivery and antenatal monitoring where consistent results are reported in a variety of settings. These effects are seen in both ‘broad’ and ‘narrow’ CCT programmes, and considering the timeframe of these programmes, the time-to-effects can be considered rapid. These results come with three major caveats: rigorous cost-effectiveness data are not available; main impact channels are not evaluated; and effects are not directly comparable across different contexts, given the varying definitions of poverty and differences on the supply side.

To build on the potential demonstrated by CCT programmes, we recommend the following for the design of both implementation and evaluation of CCTs that target MNH:

- Improve evaluation and report standardized outcomes across CCT studies
- Calculate cost-effectiveness estimates for both financial and non-financial incentives for improved maternal and newborn health
- Focus on the effectiveness and quality of services delivered on the supply side, in addition to the quantity available
- Pay attention to programme design and measure pathways for potential impact
- Modify the design to enhance MNH effects with respect to conditionalities, non-financing incentives, and infrastructural barriers
- Add supply-side strengthening conditions to CCT programmes; implement targeted supply-side interventions and track supply-side baseline and outcome levels
- Understand the link between utilization and outcomes.

## ACKNOWLEDGEMENTS

The authors would like to acknowledge the USAID Evidence Summit team and Yuna Sakuma for helpful contributions.

## References

[1] Hogan MC, Foreman KJ, Naghavi M, Ahn SY, Wang M, Makela SM (2010). Maternal mortality for 181 countries, 1980–2008: a systematic analysis of progress towards Millennium Development Goal 5. Lancet.

[2] Darmstadt GL, Bhutta ZA, Cousens S, Adam T, Walker N, de Bernis L, Lancet Neonatal Survival Steering Team (2005). Evidence-based, cost-effective interventions: how many newborn babies can we save?. Lancet.

[3] United Nations Children's Fund (2007). The state of the world's children 2008: child survival.

[4] Barros AJD, Ronsmans C, Axelson H, Loaiza E, Bertoldi AD, França GVA (2012). Equity in maternal, newborn, and child health interventions in countdown to 2015; a retrospective review of survey data in countdown to 2015: a retrospective review of survey data from 54 countries. Lancet.

[5] World Bank (2012). World development indicators 2012.

[6] Ensor T, Cooper S (2004). Overcoming barriers to health service access: inﬂuencing the demand side. Health Policy Plan.

[7] Fiszbein AN Schady N Ferreira FHG Grosh M Keleher N Olinto P Conditional cash transfers: reducing present and future poverty. Washington, DC: World Bank, 2009. 361 p. (Policy research report no. 47603).

[8] Witter S, Khadka S, Nath H, Tiwari S (2011). The national free delivery policy in Nepal: early evidence of its effects on health facilities. Health Policy Plan.

[9] Eichler R, Levine R, the Performance-Based Incentives Working Group (2009). Performance incentives for global health: potential and pitfalls.

[10] Health Systems 20/20 (2010). Paying for performance: the *Janani Suraksha Yojana* Program in India.

[11] Gaarder M, Amanda G, Todd J (2010). Conditional cash transfers and health: unpacking the causal chain. J Dev Effect.

[12] Morris SS, Flores R, Olinto P, Medina JM (2004). Monetary incentives in primary health care and effects on use and coverage of preventive health care interventions in rural Honduras: cluster randomised trial. Lancet.

[13] de Brauw A Peterman A Can conditional cash transfers improve maternal health and birth outcomes? Evidence from El Salvador's Comunidades Solidarias Rurales. Washington, DC: International Food Policy Research Institute, 2011. 36 p. (IFPRI discussion paper no. 01080). 10.1002/hec.4012 PMC7383823 32124543

[14] Powell-Jackson T, Hanson K (2012). Financial incentives for maternal health: impact of a national programme in Nepal. J Health Econ.

[15] Lim SS, Dandona L, Hoisington JA, James SL, Hogan MC, Gakidou E (2010). India's *Janani Suraksha Yojana*, a conditional cash transfer programme to increase births in health facilities: an impact evaluation. Lancet.

[16] Barber SL, Gertler PJ (2009). Empowering women to obtain high quality care: evidence from an evaluation of Mexico's conditional cash transfer programme. Health Policy Plan.

[17] Amarante V Manacorda M Miguel E Vigorito A Do cash transfers improve birth outcomes? Evidence from matched vital statistics, social security and program data. Cambridge, MA: National Bureau of Economic Research, 2011. 47 p. (NBER working paper no. 17690).

[18] Triyana M (2012). Do health care providers respond to demand-side incentives? Evidence from Indonesia.

[19] Barber SL, Gertler PJ (2008). The impact of Mexico's conditional cash transfer programme, *Oportunidades*, on birthweight. Trop Med Int Health.

[20] Lamadrid-Figueroa H, Ángeles G, Mroz T, Urquieta-Salomón J, Hernández-Prado B, Cruz-Valdez A (2010). Heterogeneous impact of the social programme *Oportunidades* on use of contraceptive methods by young adult women living in rural areas. J Dev Effect.

[21] Baird SJ, Garfein RS, McIntosh CT, Özler B (2012). Effect of a cash transfer programme for schooling on prevalence of HIV and herpes simplex type 2 in Malawi: a cluster randomised trial. Lancet.

[22] Stecklov G, Winters P, Todd J, Regalia F (2007). Unintended effects of poverty programmes on childbearing in less developed countries: experimental evidence from Latin America. Popul Stud (Camb).

[23] Mazumdar S Mills A Powell-Jackson T Financial incentives in health: new evidence from India's *Janani Suraksha Yojana*. 2011. 47 p. (Working papers series). (http://papers.ssrn.com/sol3/papers.cfm?abstract_id=1935442, accessed on 25 November 2013). 10.1016/j.jhealeco.2015.07.001 26302940

[24] Hernandez B Ramirez D Moreno H Laird N Evaluacion del impacto de *Oportunidades* en la mortalidad materna e infantile. *In:* Resultados de la. Evaluacion Externa del Programa de Desarrollo Humano Oportunidades. Morelos: Instituto Nacional de Salud Pública, 2003.

[25] Das A, Rao D, Hagopian A (2011). India's *Janani Suraksha Yojana*: further review needed. Lancet.

[26] Devadasan N Elias MA John D Grahacharya S Ralte L A conditional cash assistance programme for promoting institutional deliveries among the poor in India: process evaluation results. *In*: Richard F, Witter S, De Brouwere V, editors. Reducing financial barriers to obstetric care in low-income countries. Antwerp: ITG Press, 2008:257-73.

[27] Sosa-Rubí SG, Walker D, Serván E, Bautista-Arredondo S (2011). Learning effect of a conditional cash transfer programme on poor rural women's selection of delivery care in Mexico. Health Policy Plan.

[28] Byrne A, Morgan A (2011). How the integration of traditional birth attendants with formal health systems can increase skilled birth attendance. Int J Gynaecol Obstet.

[29] Varkey LC, Mishra A, Das A, Ottolenghi E, Huntington D, Adamchak S (2004). Involving men in maternity care in India.

[30] Hussein J Kanguru L Astin M Munjanja S What kinds of policy and programme interventions contribute to reductions in maternal mortality? The effectiveness of primary level referral systems for emergency maternity care in developing countries. London: EPPI-Centre, Social Science Research Unit, Institute of Education, University of London. 48 p. (Technical report).

[31] Holmes W Kennedy E Reaching emergency obstetric care: overcoming the ‘second delay’. Melbourne: Burnet Institute on behalf of Compass, 2010. 95 p. (Briefing paper).

[32] Garcia M Moore CMT The cash dividend: the rise of cash transfer programs in sub-Saharan Africa. Washington, DC: World Bank, 413 p.

[33] Fernandez L, Olfindo R (2011). Overview of the Philippine's conditional cash transfer program: the Pantawid Pamilyang Pilipino Program (Pantawid Pamilya).

[34] Chávez F (2010). Bolivia: cash for checkups to slash maternal deaths.

[35] Gutierrez JP (2011). Evaluacion Externa de Impacto del Programa de Transferencias Monetarias Condicionadas: Mi Familia Progresa.

[36] Urquieta J, Angeles G, Mroz T, Lamadrid-Figueroa H, Hernández B (2009). Impact of *Oportunidades* on skilled attendance at delivery in rural areas. Econ Dev Cult Change.

[37] Feldman BS, Zaslavsky AM, Ezzati M, Peterson KE, Mitchell M (2009). Contraceptive use, birth spacing, and autonomy: an analysis of the *Oportunidades* program in rural Mexico. Stud Fam Plann.

